# Supplementary material for: Long-term inactivation mediated by different FGF-A homologues on heterologously expressed NaV1.2 currents
Source: J Gen Physiol. 2026 May 22;158(4):e202613985. doi: 10.1085/jgp.202613985 (PMC13196787; doi:10.1085/jgp.202613985)
Supplement: Table S1 — shows parameters determined from Boltzmann fits to GV curves for NaV1.2 alone or when coexpressed with A-type FGF isoforms. [file jgp_202613985_tables1.docx]

**Table S1. Parameters determined from Boltzmann fits to GV curves for**

**Na_V_1.2 alone or when co-expressed with A-type FGF isoforms.**

| **Constructs** | **GV curve** | | | | **Inactivation τ (0 mV)** | |  |
| --- | --- | --- | --- | --- | --- | --- | --- |
|  | **V_h_ (mV)** | **P value** | **z (*e*)** | **P value** | **τ_i_ (ms)** | **P value** | **N** |
| **Nav1.2** | **-17.1 ± 6.5** | **0.52 ^14A^ 0.85 ^13A^ 0.99 ^12A^ 0.33 ^11A^** | **4.35 ± 1.49** | **0.99 ^14A^ 0.90 ^13A^ 0.95 ^12A^ 0.99 ^11A^** | **0.36 ± 0.09** | **<0.0001^14A^ 0.25 ^13A^ 0.14 ^12A^ 0.70 ^11A^** | **14 (8)** |
| **+FGF14A** | **-20.3 ± 4.2** | **>0.99^13A^ 0.59 ^12A^ 0.95 ^11A^** | **4.47** **± 0.88** | **0.95 ^13A^ 0.86 ^12A^**  **0.99 ^11A^** | **0.58 ± 0.12** | **0.70 ^13A^ 0.049 ^12A^**  **>0.99 ^11A^** | **15 (10)** |
| **+FGF13A** | **-19.9 ± 5.7** | **0.81 ^12A^ 0.96 ^11A^** | **4.92 ± 1.87** | **0.67 ^12A^ 0.99 ^11A^** | **0.49 ± 0.10** | **0.99 ^12A^ >0.99 ^11A^** | **5 (4)** |
| **+FGF12A** | **-16.2 ± 3.5** | **0.38 ^11A^** | **3.89 ± 0.59** | **0.86 ^11A^** | **0.45 ± 0.06** | **0.99 ^11A^** | **5 (1)** |
| **+FGF11A** | **-21.1 ± 6.2** |  | **4.62 ± 0.96** |  | **0.52 ± 0.22** |  | **6 (5)** |

**For GV curves, statistical analyses were performed using one-way ANOVA followed by Tukey’s multiple comparisons test. For inactivation times constants, Welch's ANOVA followed by Dunnett's T3 multiple comparisons test was employed.**
