# Supplementary material for: Long-term inactivation mediated by different FGF-A homologues on heterologously expressed NaV1.2 currents
Source: J Gen Physiol. 2026 May 22;158(4):e202613985. doi: 10.1085/jgp.202613985 (PMC13196787; doi:10.1085/jgp.202613985)
Supplement: Table S2 — shows parameters determined from Boltzmann fits to SSI curves for NaV1.2 alone or coexpressed with A-type FGF isoforms. [file jgp_202613985_tables2.docx]

**Table S2. Parameters determined from Boltzmann fits to steady-state inactivation (SSI) curves for Na_V_1.2 alone or co-expressed with A-type FGF isoforms**

| **Constructs** | **SSI curve** | | | |  |
| --- | --- | --- | --- | --- | --- |
|  | **V_h_ (mV)** | **P value** | **z (*e*)** | **P value** | **N** |
| **Na_V_1.2** | **-62.4 ± 3.5** | **<0.0001^14A^**  **<0.0001^13A^**  **0.0001^12A^**  **0.009 ^11A^** | **4.51 ± 0.50** | **<0.0001 ^14A^**  **<0.0001 ^13A^**  **0.09 ^12A^**  **0.23 ^11A^** | **22 (16)** |
| **+FGF14A** | **-48.2 ± 2.6** | **0.94 ^13A^**  **0.22 ^12A^**  **0.007 ^11A^** | **5.85 ± 0.64** | **0.33 ^13A^**  **0.001 ^12A^**  **<0.0001 ^11A^** | **31**  **(18)** |
| **+FGF13A** | **-49.0 ± 2.3** | **0.45 ^12A^**  **0.02 ^11A^** | **6.11 ± 0.42** | **<0.0001 ^12A^**  **<0.0001 ^11A^** | **25**  **(16)** |
| **+FGF12A** | **-51.8 ± 3.7** | **0.54 ^11A^** | **5.05 ± 0.39** | **0.98 ^11A^** | **8**  **(2)** |
| **+FGF11A** | **-55.4 ± 5.1** |  | **4.91 ± 0.31** |  | **11**  **(7)** |

**Statistical analyses were performed using** **Welch’s ANOVA test followed by Dunnett’s T3 multiple comparisons test (for V_h_) or one-way ANOVA followed by Tukey’s multiple comparisons test (for z).**
