# Supplementary material for: Long-term inactivation mediated by different FGF-A homologues on heterologously expressed NaV1.2 currents
Source: J Gen Physiol. 2026 May 22;158(4):e202613985. doi: 10.1085/jgp.202613985 (PMC13196787; doi:10.1085/jgp.202613985)
Supplement: Table S3 — shows activation parameters for NaV1.2_IQM expressed alone or with FGF-A isoforms. [file jgp_202613985_tables3.docx]

**Table S3. Activation parameters for Na_V_1.2_IQM expressed alone or with FGF-A isoforms.**

| **Constructs** | **GV curve** | | | |  |
| --- | --- | --- | --- | --- | --- |
|  | **V_h_ (mV)** | **P value** | **z (*e*)** | **P value** | **N** |
| **Na_V_1.2_IQM** | **-23.1 ± 2.9** | **0.31 ^14A^**  **0.99 ^13A^**  **0.99 ^12A^**  **0.78 ^11A^** | **5.01 ± 0.66** | **0.43 ^14A^**  **>0.99 ^13A^**  **>0.99 ^12A^**  **0.06 ^11A^** | **12**  **(7)** |
| **+FGF14A** | **-26.8 ± 6.4** | **0.38 ^13A^ 0.49 ^12A^**  **0.08 ^11A^** | **4.26 ± 0.69** | **0.34 ^13A^**  **0.45 ^12A^**  **>0.99 ^11A^** | **5**  **(5)** |
| **+FGF13A** | **-22.5 ± 1.7** | **0.99 ^12A^ 0.97 ^11A^** | **5.13 ± 0.44** | **0.99 ^12A^**  **0.20 ^11A^** | **4**  **(4)** |
| **+FGF12A** | **-23.4 ± 3.3** | **0.76 ^11A^** | **4.96 ± 0.39** | **0.049 ^11A^** | **7**  **(5)** |
| **+FGF11A** | **-21.1 ± 2.3** |  | **4.39 ± 0.09** |  | **6**  **(4)** |

**Statistical analyses were performed using** **one-way ANOVA followed by Tukey’s multiple comparisons test (for V_h_) or Welch’s ANOVA test followed by Dunnett’s T3 multiple comparisons test (for z).**
