# Supplementary material for: Long-term inactivation mediated by different FGF-A homologues on heterologously expressed NaV1.2 currents
Source: J Gen Physiol. 2026 May 22;158(4):e202613985. doi: 10.1085/jgp.202613985 (PMC13196787; doi:10.1085/jgp.202613985)
Supplement: Table S4 — shows Boltzmann fit parameters to SSI curves for NaV1.2_IQM alone or when coexpressed with A-type FGF isoforms. [file jgp_202613985_tables4.docx]

**Table S4. Boltzmann fit parameters to SSI curves for Na_V_1.2_IQM alone**

**or when co-expressed with A-type FGF isoforms.**

| **Constructs** | **SSI curve** | | | |  |
| --- | --- | --- | --- | --- | --- |
|  | **V_h_ (mV)** | **P value** | **z (*e*)** | **P value** | **N** |
| **Na_V_1.2_IQM** |  |  |  |  |  |
| **+FGF14A** | **-44.4 ± 1.4** | **0.03 ^13A^**  **0.84 ^12A^**  **0.94 ^11A^** | **7.46 ± 1.05** | **0.30 ^13A^ 0.84 ^12A^**  **0.69 ^11A^** | **5**  **(5)** |
| **+FGF13A** | **-40.8 ± 2.7** | **0.06 ^12A^**  **0.03 ^11A^** | **8.64 ± 1.29** | **0.03 ^12A^ 0.02 ^11A^** | **6**  **(4)** |
| **+FGF12A** | **-43.5 ± 2.3** | **0.99 ^11A^** | **6.97 ± 1.04** | **0.99 ^11A^** | **10**  **(6)** |
| **+FGF11A** | **-43.8 + 1.2** |  | **6.81 ± 1.03** |  | **10**  **(4)** |

**Statistical analyses were performed using one-way ANOVA followed by Tukey’s multiple comparisons test.**
