# Supplementary material for: Long-term inactivation mediated by different FGF-A homologues on heterologously expressed NaV1.2 currents
Source: J Gen Physiol. 2026 May 22;158(4):e202613985. doi: 10.1085/jgp.202613985 (PMC13196787; doi:10.1085/jgp.202613985)
Supplement: Table S5 — shows effects of FGF-A homologues on NaV1.2 and NaV1.2_IQM GV curves, SSI, and taus. [file jgp_202613985_tables5.docx]

**Table S5. Effects of FGF-A homologues on Na_V_1.2 and Na_V_1.2_IQM GV curves, SSI and taus**

|  | **GV curves** | | | | | | |  |
| --- | --- | --- | --- | --- | --- | --- | --- | --- |
| **Constructs** | **WT** | **IQM** |  |  | **WT** | **IQM** |  |  |
|  | **V_h_ (mV)** | | **P value** |  | **z (*e*)** | | **P value** | **N** |
| **Na_V_1.2** | **-17.1 ± 6.5** | **-23.1 ± 2.9** | **0.007** |  | **4.35 ± 1.49** | **5.01 ± 0.66** | **0.17** | **14/12** |
| **+FGF14A** | **-20.3 ± 4.2** | **-26.8 ± 6.4** | **0.02** |  | **4.47 ± 0.88** | **4.26 ± 0.69** | **0.63** | **15/5** |
| **+FGF13A** | **-19.9 ± 5.7** | **-22.5 ± 1.7** | **0.42** |  | **4.92 ± 1.87** | **5.13 ± 0.44** | **0.84** | **5/4** |
| **+FGF12A** | **-16.2 ± 3.5** | **-23.4 ± 3.3** | **0.004** |  | **3.89 ± 0.59** | **4.96 ± 0.39** | **0.003** | **5/7** |
| **+FGF11A** | **-21.1 ± 6.2** | **-21.1 ± 2.3** | **0.72** |  | **4.62 ± 0.96** | **4.39 ± 0.09** | **0.56** | **6/6** |
|  | **SSI curves** | | | | | | |  |
| **Constructs** | **WT** | **IQM** |  |  | **WT** | **IQM** |  |  |
|  | **V_h_ (mV)** | | **P value** |  | **z (*e*)** | | **P value** | **N** |
| **Na_V_1.2** | **-62.4 ± 3.5** | **-** |  |  | **4.51 ± 0.50** | **-** |  | **22/na** |
| **+FGF14A** | **-48.2 ± 2.6** | **-44.4 ± 1.4** | **0.003** |  | **5.85 ± 0.64** | **7.46 ± 1.05** | **<0.0001** | **31/5** |
| **+FGF13A** | **-49.0 ± 2.3** | **-40.8 ± 2.7** | **<0.0001** |  | **6.11 ± 0.42** | **8.64 ± 1.29** | **<0.0001** | **25/6** |
| **+FGF12A** | **-51.8 ± 3.7** | **-43.5 ± 2.3** | **<0.0001** |  | **5.05 ± 0.39** | **6.97 ± 1.04** | **0.0001** | **8/10** |
| **+FGF11A** | **-55.4 ± 5.1** | **-43.8 ± 1.2** | **<0.0001** |  | **4.91 ± 0.31** | **6.81 ± 1.03** | **<0.0001** | **11/10** |
|  | **Onset of Inactivation at 0 mV** | | |  | **Recovery from Inactivation at -80 80808088080 mV** | | |  |
| **Constructs** | **WT** | **IQM** |  |  | **WT** | **IQM** |  |  |
|  | **T_i_ (ms)** | | **P value** |  | **T_slow_ (ms)** | | **P value** | **N** |
| **Na_V_1.2** | **0.36 ± 0.09** | **-** |  |  | **-** | **-** |  | **22/na** |
| **+FGF14A** | **0.58 ± 0.12** | **0.92 ± 0.24** | **0.0004** |  | **592.9 ± 111.5** | **565.5 ± 88.3** | **0.64** | **31/4** |
| **+FGF13A** | **0.49 ± 0.10** | **1.50 ± 0.30** | **0.0002** |  | **2017.6 ± 324.0** | **1831.5 ± 383.5** | **0.23** | **25/6** |
| **+FGF12A** | **0.45 ± 0.06** | **1.33 ± 0.20** | **<0.0001** |  | **497.2 ± 97.7** | **565.0 ± 112.5** | **0.20** | **8/10** |
| **+FGF11A** | **0.52 ± 0.22** | **0.88 ± 0.16** | **0.009** |  | **173.1 ± 19.7** | **155.8 ± 34.5** | **0.17** | **11/10** |

**Statistical analyses between the two groups (WT vs IQM) were performed using unpaired t test.**
