# Supplementary material for: Long-term inactivation mediated by different FGF-A homologues on heterologously expressed NaV1.2 currents
Source: J Gen Physiol. 2026 May 22;158(4):e202613985. doi: 10.1085/jgp.202613985 (PMC13196787; doi:10.1085/jgp.202613985)
Supplement: Table S6 — shows use-dependent changes in FGF-A–mediated recovery from LTI. [file jgp_202613985_tables6.docx]

**Table S6. Use-dependent changes in FGF-A-mediated recovery from LTI**

| **10-Pulse Train frequencies** | **w/o FGF-A** | | | | | |
| --- | --- | --- | --- | --- | --- | --- |
|  | **τ_f_ (ms)** | **A_f_** | **τ_s_ (ms)** | **A_s_** |  | **N** |
| **1P** | **8.67 ± 2.27** | **0.99 ± 0.02** | **-** | **-** |  | **22/0** |
| **5 Hz** | **8.27 ± 3.24** | **0.95 ± 0.03** | **97.3 ± 41.0** | **0.06 ± 0.01** |  | **3/6** |
| **10 Hz** | **8.99 ± 3.19** | **0.94 ± 0.03** | **153.0 ± 59.8** | **0.05 ± 0.03** |  | **3/6** |
| **20 Hz** | **9.29 ± 2.89** | **0.91 ± 0.05** | **105.6 ± 39.2** | **0.12 ± 0.03** |  | **3/6** |
| **40 Hz** | **10.23 ± 3.67** | **0.90 ± 0.05** | **131.2 ± 29.4** | **0.09 ± 0.03** |  | **4/5** |
|  | **+ FGF14A** | | | | | |
| **1P** | **5.38 ± 1.79** | **0.40 ± 0.06** | **592.9 ± 111.5** | **0.60 ± 0.06** |  | **31** |
| **2 Hz** | **6.13 ± 2.91** | **0.23 ± 0.05** | **648.6 ± 34.0** | **0.74 ± 0.08** |  | **2** |
| **5 Hz** | **6.62 ± 2.16** | **0.16 ± 0.04** | **662.9 ± 83.9** | **0.83 ± 0.04** |  | **9** |
| **10 Hz** | **8.66 ± 2.38** | **0.11 ± 0.04** | **629.1 ± 78.3** | **0.86 ± 0.03** |  | **8** |
| **20 Hz** | **9.49 ± 3.39** | **0.09 ± 0.03** | **620.4 ± 70.3** | **0.91 ± 0.06** |  | **8** |
| **40 Hz** | **12.07 ± 8.22** | **0.07 ± 0.04** | **635.1 ± 68.7** | **0.91 ± 0.04** |  | **8** |
|  | **+ FGF13A** | | | | | |
| **1P** | **6.31 ± 1.74** | **0.54 ± 0.06** | **2017.6 ± 324.0** | **0.45 ± 0.06** |  | **25** |
| **5 Hz** | **15.33 ± 12.66** | **0.17 ± 0.06** | **2426.2 ± 394.2** | **0.79 ± 0.06** |  | **5** |
| **10 Hz** | **5.66** | **0.14** | **1614** | **0.77** |  | **1** |
| **20 Hz** | **9.05 ± 3.34** | **0.15 ± 0.08** | **1900.7 ± 629.5** | **0.79 ± 0.11** |  | **3** |
| **40 Hz** | **14.75 ± 9.26** | **0.10 ± 0.04** | **2230.8 ± 524.5** | **0.86 ± 0.05** |  | **5** |
|  | **+ FGF12A** | | | | | |
| **1P** | **4.96 ± 2.60** | **0.70 ± 0.04** | **497.2 ± 97.7** | **0.29 ± 0.04** |  | **8** |
| **5 Hz** | **4.69 ± 0.64** | **0.44 ± 0.07** | **633.5 ± 52.4** | **0.54 ± 0.07** |  | **5** |
| **10 Hz** | **5.42 ± 0.84** | **0.32 ± 0.08** | **602.2 ± 44.5** | **0.64 ± 0.08** |  | **5** |
| **20 Hz** | **7.25 ± 2.25** | **0.24 ± 0.09** | **592.2 ± 81.6** | **0.74 ± 0.09** |  | **5** |
| **40 Hz** | **8.64 ± 1.72** | **0.19 ± 0.10** | **540.5 ± 53.6** | **0.77 ± 0.10** |  | **5** |
|  | **+ FGF11A** | | | | | |
| **1P** | **7.92 ± 2.73** | **0.55 ± 0.05** | **173.1 ± 19.7** | **0.44 ± 0.05** |  | **11** |
| **5 Hz** | **8.28 ± 2.39** | **0.45 ± 0.04** | **230.8 ± 39.2** | **0.51 ± 0.04** |  | **5** |
| **10 Hz** | **9.61 ± 2.02** | **0.39 ± 0.05** | **239.0 ± 19.3** | **0.58 ± 0.04** |  | **5** |
| **20 Hz** | **10.04 ± 3.02** | **0.32 ± 0.04** | **227.2 ± 11.7** | **0.65 ± 0.05** |  | **5** |
| **40 Hz** | **10.93 ± 5.85** | **0.29 ± 0.03** | **201.0 ± 56.3** | **0.68 ± 0.04** |  | **5** |
